# Developing historical thinking skills and creativity of visually impaired middle school students

**DOI:** 10.3389/fpsyg.2025.1509297

**Published:** 2025-06-18

**Authors:** Osman Akhan, Adem Uzun

**Affiliations:** ^1^Faculty of Education, Akdeniz University, Antalya, Türkiye; ^2^Faculty of Education, Cumhuriyet University, Sivas, Türkiye

**Keywords:** historical thinking skills, creativity, visually impaired, middle school, history education, action research

## Abstract

Since visually impaired students in Türkiye are subject to the general education curriculum, they may experience difficulties with the topics and activities included in the existing program. Especially in a subject like history, which involves abstract concepts, students can overcome these challenges more easily through activities specifically designed for them. In this context, the aim of the study is to develop the historical thinking skills and creativity of a group of visually impaired middle school students within the scope of the 5th grade “Journey to History” unit. The study is a qualitative research designed as an action research. The study group of the research consisted of 14 visually impaired 5th grade students, 5 girls and 9 boys, aged between 10 and 12, studying at a middle school for the visually impaired in Turkey in the 2022–2023 academic year. The data of the study were collected through face-to-face interviews with the students during the stages of the action research. The data obtained were transferred to the MAXQDA 2020 Plus qualitative data analysis program and analyzed with the descriptive analysis method and themes were created. According to the findings of the study, visually impaired students had the opportunity to develop their historical thinking skills and creativity. Students gained a deeper understanding of historical periods, chronological concepts and historical events. In particular, the processes that students learned by touching and feeling during museum activities improved their historical empathy and historical analysis skills. In addition to historical thinking skills, the activities also strengthened students’ creative thinking skills. Within this process, the students had been informed about the ancient civilizations of Anatolia and their historical contexts, and these pieces of information had been expressed by creative writings and animations. Students gained a significant improvement in historical thinking and creativity skills. Braille timelines and museum activities made it possible for students to comprehend abstract history concretely. It was an effective example to reduce the difficulties faced in front of visually impaired students while studying history. This study aims to emphasize the importance of accessible teaching practices that support the historical thinking and creativity skills of visually impaired students, while also contributing to both the curriculum and teacher training for inclusive history education.

## 1 Introduction

One of the goals of developing countries in the 21st century is to establish a high-quality education system. The quality of the National Curriculum developed and implemented determines the success of a country’s education system and the value of its human capital. The aim of the National Curriculum is to raise young generations skilled in 21st-century competencies. Students who master these skills will possess critical, creative, and innovative thinking abilities and will be able to compete on an international level. In this context, education is the cornerstone of every society and should be accessible to all student groups, including those with special needs and disadvantaged groups. One such group is visually impaired students. The primary goal of education for all student groups is to develop a sense of belonging to their respective communities. History lessons play a crucial role in fostering this sense of belonging.

History lessons typically consist of verbal information such as objects, people, wars, treaties, terms, places, dates, classifications, and stories, which explain what an event is and why it happened. For many years, history lessons have been shaped by a memorization method, where students were expected to repeat and summarize the verbal information presented in notes or books. However, the verbal structure of history lessons and the preferred teaching method have led to the subject becoming boring. As a result, students have gradually perceived history as a collection of past stories that are not useful for their future and academic development, leading to a loss of interest in the subject (Black and Blake, 2001; Jensen, 2001; Zhao and Hoge, 2005; Şimşek, 2008; López-Fernández et al., 2023). In this context, there is a need for new teaching strategies in history education to enhance students’ motivation and academic success in history classes. To make history teaching more effective, lessons can be planned in line with historical thinking skills.

### 1.1 Historical thinking skills

As a result of the studies conducted on history teaching in the last 50 years, researchers have determined that history teaching is rote-learning-based (Pan et al., 2023; Fertig, 2005; Levstik and Barton, 2005; VanSledright, 2010), textbook-oriented (Hicks, 2004; Miralles et al., 2014; Callison, 2013; De La Paz, 2013), and teacher-centered (Schug et al., 1984; Cuban, 1993; Serkan and Yiğit, 2017; Akhan, 2022). This teaching model has transformed history classes into dull, rote, and disliked subjects (Jensen, 2001; Black and Blake, 2001; Zhao and Hoge, 2005; Alaca, 2019). In this regard, it can be said that traditional approaches to history teaching reduce students’ interest in the subject, making the process of learning history ineffective and unmotivating.

In the light of the data revealed by the researchers, the paradigm of history education has changed, and history education has also changed with the new understanding of history teaching developed in the last quarter of the twentieth century. Historical thinking has become one of the main goals of history education in many countries, requiring students to understand how history is structured as a discipline as well as the ability to understand the past (Wilke and Depaepe, 2019; Ofianto, 2021; Martínez-Hita et al., 2022). In this context, history education has evolved beyond merely transmitting information, aiming instead to cultivate individuals who, through historical thinking skills, can question and make sense of the past while establishing interdisciplinary connections.

Historical thinking skills, which have changed the paradigm of history education, can be described as a set of advanced thinking skills that includes researching and thinking like historians (Culminas-Colis et al., 2016; Van Drie and Van Boxtel, 2008; VanSledright and Limón, 2006), analyzing historical events and concepts within their unique contexts (Reisman and Wineburg, 2008; Ofianto, 2021; Lee and Ashby, 2000; De Leur et al., 2017) relating past events to current ones (Dilek, 2007; Santi and Reed, 2015; Levstik and Barton, 2015; Lee, 2023), using primary sources (VanSledright and Kelly, 1998; Wineburg, 2001; Drake and Brown, 2003; Seixas and Morton, 2013), conducting historical inquiry (Lee, 1983; Tally and Goldenberg, 2005; Reisman, 2012; Talin, 2015; López-Fernández et al., 2023), and developing critical, (Levstik and Barton, 2005; De La Paz, 2013; Seixas, 2017; Ofianto and Suhartono, 2016), creative (Wineburg, 2001; Cowgill and Waring, 2017; Douma, 2018), and analytical thinking (Sokolov, 2015; Seixas, 2017; Gibson and Peck, 2020; Keleşzade et al., 2018), decision making (Halpern, 2007; Retnawati et al., 2018; Culminas-Colis et al., 2016; Cowgill and Waring, 2017), as well as historical empathy (Endacott and Brooks, 2013; Rantala, 2011; Seixas and Peck, 2004; Levesque, 2008), perspective (Levstik and Barton, 2001; Savich, 2008; Seixas and Morton, 2013; Lee, 2023), and chronological reasoning (UCLA, 2024; Demircioğlu, 2009; Lorenc et al., 2013). Within this framework, historical thinking skills not only aim to understand the past but also offer a holistic learning approach that enables students to interpret history with a critical, analytical, and creative perspective, helping to shape them into active, inquisitive, and conscious individuals.

### 1.2 How to teach historical thinking skills?

History cannot be lived, directly observed, or reproduced. As a result of the answers sought to the question of when historical thinking skills should be taught in history education, which is full of abstract concepts, Chowen (2005) concluded in his research that students can learn historical thinking from the beginning of history education. VanSledright (1999) states that elementary school students can think historically with appropriate guidance and practice, while Levstik and Barton (1996) argued that even six-year-old students can engage in historical thinking. Additionally, Barton (1997) concluded that fourth-grade students might have the ability to carefully examine primary sources.

Historical thinking in students, whom we believe can acquire these skills at a young age, does not emerge easily or naturally (Wineburg, 2001). History teachers have a crucial role in imparting these skills to students. Teachers need to be knowledgeable about various instructional strategies and use well-designed teaching materials to reveal and develop these skills in learners (Van Drie and Van Boxtel, 2008; Santisteban Fernández, 2010; Endacott, 2010; Savenije and De Bruijn, 2017). Thus, Historical Thinking Skills help students explore complex ideas and abstract concepts through cognitive processes with the guidance of teachers. Through these skills, students learn the way historians use a real source to reconstruct past events and develop their skills to understand the history, characters, events, places and activities of the human past to determine the significance of a history.

History education helps individuals make sense of the past and develop critical thinking skills. However, fostering historical thinking skills requires different approaches for each student group. Some groups, in particular, may face challenges in acquiring these skills due to accessibility barriers in education. In this respect, enabling visually impaired students to develop historical thinking skills is not only an educational challenge but also carries great importance in terms of making education inclusive. The difficulties faced by visually impaired students in Türkiye further highlight the necessity of making history education accessible. Teaching history to visually impaired students requires the use of specialized teaching methods and approaches to support the development of their historical thinking skills.

### 1.3 Visually impaired individuals and history education

Education is one of the fundamental rights expected to be provided to all individuals in society according to their needs. For individuals with disabilities to actively participate in society, meet their personal needs, and continue their lives without requiring assistance from others, their right to education is of vital importance. As a matter of fact, national and international authorities, aware of this importance, have tried to guarantee the right to education of individuals with the conventions and protocols they have put into practice. International agreements such as the Universal Declaration of Human Rights (UDHR, 1948) and the European Convention on Human Rights (ECHR, 1950) are examples of protocols and treaties established to safeguard the right to education. However, visually impaired individuals, being a disadvantaged group, often do not benefit sufficiently from education due to economic, socio-cultural, and religious factors. In the globalized world, visually impaired individuals are at a greater disadvantage compared to their peers in accessing information and learning (Smith et al., 2004; Salleh and Zainal, 2010; Zebehazy et al., 2012). It is crucial to address this disadvantage and integrate visually impaired students into society.

While all sensory organs are important for learning, the eye is considered the most crucial due to the rich information it provides (Ataman, 2003), with 80–85% of the information acquired during learning coming through vision (Cavkaytar and Diken, 2012; Ataman, 2012). İn this context, it can be said that vision plays a significant role in individuals’ learning, and any impairment in the sense of sight can affect the student’s learning level. For visually impaired individuals, touch and hearing are often the primary senses involved in learning (D’Agnano et al., 2015). However, since learning in school is largely dependent on vision, students with this disability frequently experience academic difficulties (Kumar et al., 2001). Therefore, in order to minimize the challenges faced by visually impaired students in the learning process, it becomes essential to develop alternative and effective teaching methods that engage their other senses, such as hearing and touch.

Visually impaired students face numerous challenges in participating in history classes and understanding historical events. The lack of vision is a significant barrier to accessing historical sources and interpreting visual materials. For the visually impaired, history is a gateway to trace the past, to understand societal developments and to connect themselves with stories from the deep past. However, their access to historically presented information, which is traditionally delivered through visual materials, is limited. Therefore, developing historical thinking skills for visually impaired individuals requires alternative methods and technologies.

For visually impaired individuals, auditory and tactile materials (Bishop, 1997; Palmer, 2005; Okcu et al., 2022; Lyndon Gillon and Young, 2002; Şahin and Yörek, 2009) are of great importance. Historical texts written in Braille, audiobooks, tactile museums, music, and similar resources are crucial (Al-Aref et al., 2022). These materials enable visually impaired individuals to grasp historical events in a more concrete manner. Additionally, studies on the education of visually impaired students have identified the need for a flexible curriculum, adequate materials, differentiated teaching methods, diverse and continuous experiences, and a rich learning environment with appropriate assessment procedures (Koenig and Holbrook, 2000; Simon et al., 2010; Bayram et al., 2015; Kapucu and Kızılaslan, 2022). Therefore, in order for visually impaired students to succeed in their history learning processes, it is crucial to create a flexible, inclusive educational environment that is responsive to individual needs, along with materials that support sensory diversity.

Upon reviewing the literature, only one source (Al-Aref et al., 2022) specifically addresses the teaching of history to visually impaired individuals. This study aimed to examine the perspectives of visually impaired students regarding history lessons in middle school. The findings of the research indicated that history lessons are not sufficiently accessible to visually impaired students. Based on this need, the aim of this study is to conduct a practical study on history teaching for visually impaired students and to show activities that can be an example for teachers/researchers. In this direction, this study aims to contribute to the creativity of a group of visually impaired middle school students by developing their historical thinking skills (one of the effective ways in history teaching) within the scope of the 5th grade Journey to History unit.

## 2 Methodology

### 2.1 Research model

This study is a qualitative research designed as action research. Action research “improves teaching practices, increases interest in classrooms and studies, and works to improve oneself” (Arora, 2017). There are different action research approaches. In this research, “school wide action research” was used. In this action research process, teachers/researchers can choose to focus their work on one student, a small group of students, one/several classes, or a school. School-wide action research is a school reform initiative and its results can lead to school-wide change (Hewitt and Little, 2005). In this study, it was aimed to develop the historical thinking skills of a group of visually impaired middle school students within the scope of the subject of Anatolian and Mesopotamian civilizations in the culture and heritage unit of the social studies course.

### 2.2 Study group

The study group of this research consists of 14 visually impaired 5th-grade students, aged 10–12, enrolled in a visually impaired middle school in Turkey during the 2022–2023 academic year. The reason for selecting 5th grade students in the study is that the topic of Anatolian and Mesopotamian civilizations, chosen to develop historical thinking skills, is included in the 5th grade Social Studies curriculum under the “Culture and Heritage” unit. Additionally, at the time the study was conducted, only 14 students at the 5th grade level could be reached at the visually impaired middle school. Therefore, the 14 students who volunteered to participate were selected as the study group.

Among these students, 5 are children with congenital total blindness. Three of the students have congenital conditions with 10% vision, including eye pressure and Bardet-Biedl Syndrome. Two students have 30% vision due to illnesses in infancy, such as nephrotic syndrome and eye pressure. The remaining four students lost their vision completely due to conditions affecting the head in childhood, such as brain tumors, eye cancer, and eye pressure. These students in the study group stay with their families and attend a middle school that provides education for them. Consent forms have been obtained from the parents of the students involved in the study.

### 2.3 Data collection and analysis

The data for this research were collected through face-to-face interviews with students during the action research process. Additionally, written works created by visually impaired students using Braille were read by a teacher at the school for visually impaired and made available for the researchers’ use. In addition, the activities with visually impaired students were audio-recorded and the researchers alternately took notes of the answers given. All the data obtained were transferred to the MAXQDA 2020 Plus qualitative data analysis program and analyzed with the descriptive analysis method and themes were created. In addition, the findings were supported by visualizing and presenting the codes containing the students’ explanations in a clear and concise way and by direct quotations (1FS: 1st female student, 2MS: 2nd male student).

For the reliability of the study, the analyses were conducted simultaneously by two researchers and an external researcher in different locations. For the reliability of the study, the formula R (Reliability) = [Na (Agreement)/Na (Agreement) + Nd (Disagreement)] × 100 as suggested by Miles and Huberman (1994) was used. According to the calculation, the inter-rater reliability was calculated as 94% and the analysis of the research was accepted as reliable.

The action phase process, which was preferred as the method in the research, was carried out in 4 stages. In the 1st stage, the action planning stage, firstly, Social Studies teachers were interviewed. Teachers were asked about the subjects they had difficulty in teaching their lessons and the subjects that students had difficulty in understanding. According to these interviews, teachers stated that students had difficulty in history topics in the culture and heritage unit. They stated that the students had difficulty in explaining the outcome “SB.5.2.1. Recognizes the important contributions of Anatolian and Mesopotamian civilizations to human history based on their tangible remains” (Ministry of National Education, 2023a) in the 5th grade Culture and Heritage unit every year. In addition, the study group finished this subject and they could not succeed in the exam in the semester they were in this subject. In line with the students’ low grades in this subject and the teachers’ statements, it was decided to show an alternative sample application in which historical thinking skills were used.

In the application phase of the Stage 2 plan, the researchers first created an activity calendar. At the beginning and end of this activity calendar, a 10-day process was prepared with interviews and evaluation meetings with the students in the study group. At this stage, the researchers carried out activities with the students in the study group in their classrooms and in the Museum of Anatolian Civilizations. These activities were carried out by the researchers in collaboration with the teachers and support staff of the visually impaired middle school. Prior to the implementation, an introductory activity was held, followed by individual and group discussions to help students adjust to the researchers and the study process. During these discussions, topics such as introducing themselves, their interest in Social Studies and history subjects, and their future dreams were selected. Additionally, all questions from the students related to the research topic were answered directly by the researchers at the beginning of the study.

Data collection and analysis step, action planning step, putting the plan into action step and the last step (evaluation) were carried out in the third action step of the research. In the fourth action step of the study, the final action evaluation step, face-to-face interviews were conducted with the students in the study group and they were asked to evaluate the application process.

### 2.4 Application process

The applications started with introductory activities with the visually impaired students in the study group. In these activities, which were carried out with creative drama activities, it was aimed for the students to achieve achievements such as becoming open to communication, being compatible with the group, and being aware of the study goals.

The applications were carried out in two different venues, and the introductory activities and the topics of the concept of age, the history strip and Mesopotamian civilizations were carried out in the middle school for the visually impaired where the students study. The Anatolian Civilizations topic was carried out at the Museum of Anatolian Civilizations, which hosts numerous artifacts from the prehistoric period of Anatolia.

All activities in the application process were prepared and applied by the researchers. In the process of preparing the activities, the researchers received opinions from educators specialized in the field of special education for the visually impaired. In addition, the teachers of the students in the study group were consulted before the application and the whole process was carried out under the observation of the teachers. Explanations about the application process are shown in Table 1.

**Table 1:** Application process.

Process	Course subject	Course outcomes	Course content and skills to be taught
First Session^*^	Preliminary Meeting and Introduction Activities	Students interact and communicate through the drama method.	In this lesson, drama method is used to help students communicate with researchers and their peers.
Session 1^**^	Concepts of era, century, historical period, BC, AD	Students learn basic concepts	Students recognize concepts through drama and gain chronological thinking skills from historical thinking skills.
Session 2			
Session 3	Timeline	Students illustrate basic concepts on the timeline.	Students identify and provide examples of the concepts they have learned on a tactile timeline, in line with their chronological thinking skills.
Session 4			
Session 5	Mesopotamian Civilizations	Students understand the civilizations of the Sumerians, Babylonians, and Assyrians, as well as their historical significance.	Students first learn about the locations of Mesopotamian civilizations with the help of a tactile map. Efforts were made to develop their historical understanding skills regarding the historical legacy left by these civilizations.
Session 6		Students learn about cuneiform writing, tablets, ziggurats, the Code of Hammurabi, and Assyrian trade colonies.	
Session 7	Anatolian Civilizations	Students become familiar with the Hittite civilization and understand its historical significance.	Students are encouraged to develop historical analysis and interpretation skills regarding the annual (calendar), polytheistic religion, and the Treaty of Kadesh. Historical understanding skills have been developed by explaining the Hittites’ religious beliefs and social structures through Hittite myths.
Session 8			
Session 9		Students become familiar with the Urartian civilization and understand its historical significance.	Students are encouraged to develop historical understanding skills regarding the castle, water channels, and dams in Tuşpa.
Session 10		Students become familiar with the Phrygian civilization and understand its historical significance.	Students are encouraged to develop historical understanding skills regarding the fibula, the homeland of Midas, and aspects of agriculture, animal husbandry, mining, weaving, and furniture-making.
Session 11		Students become familiar with the Lydian civilization and understand its historical significance.	Students are encouraged to develop historical problem analysis and decision-making skills regarding the Royal Road and Lydian currency.
Session 12,13,14	Anatolian Civilizations Museum Activities		
Final Session^*^	Evaluation		

* For the first and last sessions, one class period was allocated, and approximately 20-min one-on-one interviews were conducted with each student.

** The average duration of the sessions for the activities was 1 h.

In Table 1, the historical thinking skills aimed to be developed during the application process are those listed on the ‘History’ section of the University of California, Los Angeles (UCLA) website. These include: “*Chronological Thinking Skills, Historical Comprehension Skills, Historical Analysis and Interpretation Skills, Historical Research Skills, and Historical Problem Analysis and Decision-Making Skills.”* In this study, in addition to the 5 skills summarized and translated from the NCHS standards in the (Ministry of National Education, 2023b) History Curriculum, historical empathy skills were also included. However, due to the age level of the study group, historical research skills were not utilized. The activities during the application process were developed to foster these skills. Additionally, in all activities, efforts were made to develop the historical thinking skill of “historical empathy.”

#### 2.4.1 Activities designed to develop the “chronological thinking skills” and creativity provided in the 1st, 2nd, 3rd, and 4th sessions of the application

For visually impaired students, sensory diversity is an important factor in the concept learning process (Kızılaslan and Sözbilir, 2018). In this regard, Merrill and Tennyson's (1977) concept teaching strategy was used in the instruction of concepts, utilizing the analogy method as well. First, definitions of historical time concepts were provided, and basic information about these concepts was given. The concept of era was concretized by relating it to stages such as infancy, childhood, adolescence, adulthood, and old age. The concept of century was related to age milestones such as first year, second year, and so on. By encouraging children to consider the most significant event in their lives as their own personal milestone, this event was related to the concepts of BC and AD. The concepts of prehistoric periods and historical eras were related to learning to write in Braille, thus aiming to concretize these concepts. Thus, it was demonstrated that they could record their thoughts, feelings, and experiences in writing, and that all recorded events could be learned by others. An analogy was made by expressing that they entered historical eras through writing. Additionally, various objects representing the Stone Age and the Bronze Age, prepared by researchers in different forms, were brought into the classroom. Students were encouraged to learn by touching and feeling these items, which provided examples of prehistoric periods. The transformation of a rough stone into a useful tool and the processing of raw metal into various objects and tools were used to develop skills in change and continuity, as well as chronological thinking.

Additionally, examples of concepts and non-examples were provided. Time periods related to BC and AD were given, and exercises were conducted on calculating time spans and identifying which era they belong to. Students prepared timelines to develop their chronological thinking skills. A time capsule activity (an adapted version) was used to develop historical empathy skills. Students were asked to imagine living in the year 2000 BC and to create a time capsule. They brainstormed to decide what to use as the capsule and which materials to include in it. It was decided that the time capsule, which is expected to be opened in AD 2023, would be a box. The capsule was to contain a stone axe, jewelry, pottery, stone and metal figurines, and various items made of copper and iron. The capsule was then buried in the schoolyard by the researchers. Afterwards, the time capsule is excavated from its buried location with the students, and the items inside are evaluated from a contemporary perspective to develop skills in change and continuity. Additionally, the duration for which the capsule was buried is calculated by noting that it was buried from 3300 BC to AD 2023. Thus, through various examples and exercises, learners acquire fundamental concepts such as era, century, period, BC (Before Christ), AD (Anno Domini), and others. Knowing these basic concepts is essential for developing chronological thinking skills. Chronological thinking is an indispensable tool for structuring a historical narrative and making sense of a sequence of events (Lorenc et al., 2013). Therefore, chronological thinking is fundamental to historical thinking and provides a mental framework for organizing historical thought (UCLA, 2024). The ability to think chronologically forms the backbone of history teaching.

#### 2.4.2 Activities designed to develop the “historical understanding skills” and creativity provided in the 5th and 6th sessions of the application

Information about the geographical features of the Mesopotamia region is provided to visually impaired students with the help of a relief map, aiming to enhance their imagination and creativity regarding the use of writing in the first civilization of the region, the Sumerians. The students are asked questions such as: ‘How might the process of the invention of writing have developed? Which group of people in society might have invented writing? Did the entire population know Sumerian writing? Who might have taught writing to the people? Which social group might have used writing the most? How might writing have been recognized by other civilizations? What would our lives be like if writing had never been invented?’ These questions aim to help students develop a historical perspective and enhance their creativity. Students are asked to think aloud and provide answers to the questions. Then, shapes corresponding to the letters of the Sumerian alphabet are described, along with visual and tactile examples of letters from the Latin alphabet, Cyrillic alphabet, and Braille alphabet. Students are then asked to invent an alphabet using sticks on clay dough. This activity is designed to enhance their creative skills.

Various passages about the Sumerians’ religious beliefs, economic life, cultural life, mythologies, social life, and laws are read from Sumerian tablets, which are used as primary sources by researchers. Students are then asked to make evaluations about Sumerian life through questions based on these primary sources, aiming to develop their historical understanding skills by creating a historical context.

In the continuation of the activities, the students are introduced to the Ziggurats, which are fundamental to the religious life of the Sumerians, and information is provided about their features. Then, the following questions are posed to help students develop their critical thinking and historical reasoning skills: ‘Considering the features of Ziggurats in Sumerian social life, what kind of relationship can be established between religious beliefs and the economic structure? What purposes might Ziggurats have served as storage facilities? Which fields of science might have developed in Ziggurats? After the Sumer period, during the Babylonian period, information is provided about the Code of Hammurabi as a primary source aimed at developing historical comprehension skills. Some legal provisions are read in class, and students are tasked with identifying which provisions pertain to criminal law, family law, commercial law, and public law. Additionally, students express their thoughts in their own words regarding the deterrent effect of these legal provisions on crime prevention. Students are asked to engage in historical empathy by imagining themselves in Hammurabi’s position and considering what laws they would establish on various issues. This exercise encourages their creativity, critical thinking, and the development of historical context. Students are invited to think aloud and provide answers to the questions.

In the continuation of the activities, the topic of the historical transition of Anatolia is introduced. Students are asked questions such as: ‘How might writing have come to Anatolia? What kind of relationships existed between the civilizations of Anatolia and Mesopotamia, and to what extent? What could be the role of writing in the transfer of culture?’ These questions aim to develop students’ historical reasoning skills, critical thinking abilities, and creativity. Clay tablets created by the Assyrians, found in the Kaniş Karum, are used as primary sources. Students read examples from these tablets and are asked to interpret the content of the historical texts by identifying who created them, when, how, and where, and to express their interpretations of the historical data in their own words. This activity is designed to develop their historical understanding skills.

#### 2.4.3 Activities designed to develop the “historical analysis and interpretation skills” and creativity provided in the 7th, and 8th sessions of the application

After providing general information about the Hittites, one of the most significant events in Hittite history, the Battle of Kadesh with the Egyptians, is narrated using primary sources. In Egyptian sources, the text known as the Poem of Pentaour states that the war was won by the Egyptians under the leadership of Ramses II, while Hittite sources express that the battle was won by them under the leadership of King Muwatalli II. Following the war, the articles of the Treaty of Kadesh, known as the world’s first international agreement, are read. To develop critical thinking skills, students are asked which side won the war, considering primary sources describing the conflict from both civilizations and the treaty signed at the end of the war. This approach aims to establish cause-and-effect relationships between historical events and to enhance historical analysis and interpretation skills by comparing the similarities and differences in historical texts that contain opposing viewpoints. In another activity, the articles of the Treaty of Kadesh are evaluated one by one by the students to assess whether they can develop a historical perspective. Students are then asked to use their historical reasoning skills to evaluate the reasons for the exact replica of this treaty being hung on the wall of the United Nations building.

In the continuation of the activities, aimed at developing historical empathy and critical thinking skills, an example from the Annals of II. Muršili, a primary source related to the Hittites, is read to the students. They are then asked to imagine themselves as Hittite rulers and prepare their own example of an annal. The events and narrative structure are left entirely to the students, encouraging them to enhance their creativity and establish historical context.

In the continuation of the activities, the tales of Hunter Kessi, Appu and his two sons, as well as the myths of the Lost God, the Moon that Fell from the Sky, and the Illuyanka Myth are read. Students are encouraged to use their critical thinking skills to evaluate these stories aloud, establishing their historical context and making assessments regarding the religious beliefs of the Hittites. In this activity, students discuss among themselves and attempt to interpret historical data in their own words. This activity is designed to develop their historical understanding skills.

#### 2.4.4 Activities designed to develop the “historical understanding skills” and creativity provided in the 9th, and 10th sessions of the application

**Figure 1:**
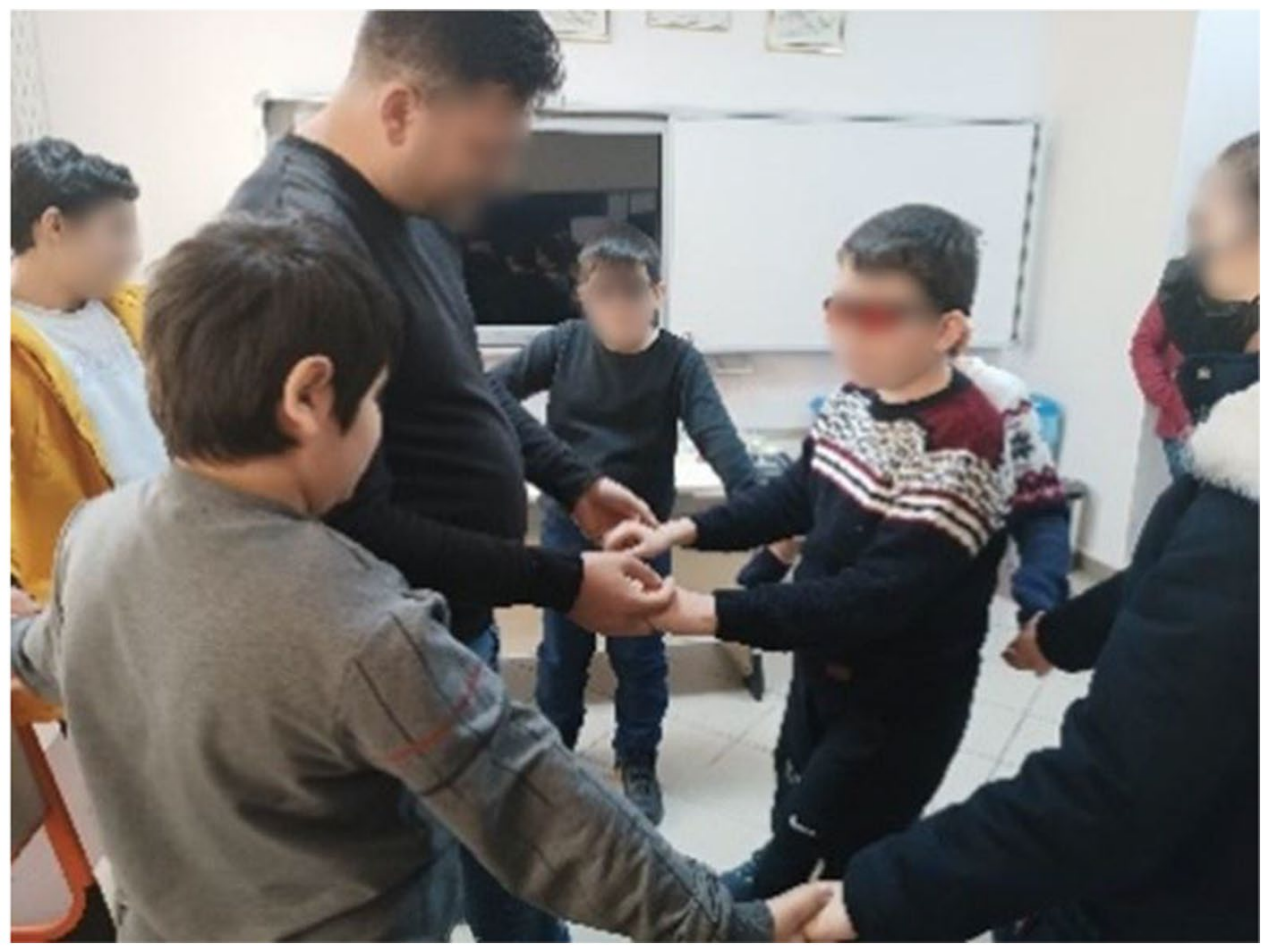
Example of in-class activities.

General information about the Urartians is provided, and students read about the water canals, dams, fortresses, and temples built during the Urartian period, using inscriptions as primary sources. The aim is for students to evaluate, using their critical thinking skills, why the Urartians placed such importance on irrigation canals and dam construction while establishing their historical context. Through this activity, students discuss among themselves and attempt to interpret historical data in their own words, thereby working to develop their historical understanding skills (Figure 1).

In the continuation of the activities, general information about the Phrygians is provided. Students are asked questions about the relationship between the region where the Phrygians were established and their economic activities (agriculture, animal husbandry, mining, weaving, furniture-making). They also discuss where the fibula (a type of brooch) might have been used and evaluate whether the laws established to improve agriculture and animal husbandry were sufficient. This encourages them to think aloud and engage in discussions. In this way, students work to develop their critical thinking skills and establish historical context. Additionally, the story of King Midas with donkey ears is read, and students are asked to act it out by taking on roles, thereby enhancing their historical empathy skills and creativity.

#### 2.4.5 Activities designed to develop the “historical problem analysis and decision-making” skills and creativity provided in the 11^th^ sessions of the application

Information is provided about the geographical region and history of the Lydians. The most significant contribution of the Lydians to world civilization is the invention of money. In this context, the following questions are posed to students to help develop their critical thinking skills, creative thinking, historical problem analysis, decision-making skills, and to establish historical context: ‘How did people conduct trade without money? Can barter be fair? How was money invented? What materials might the first money have been made from? What motifs might have appeared on the front and back of coins created by the Lydians? What criteria could determine the value of money? What might have happened in the world if money had never been invented? Is having little money or a lot of money a problem?’ Students are encouraged to think aloud, answer the questions, and discuss them with a partner. Then, following the instructions given to visually impaired students, they are asked to perform a role-play that illustrates the process leading from barter to the invention of money. This aims to help students establish historical context while developing their creative thinking and historical empathy skills.

In the continuation of the activities, information about the Royal Road is provided, and the following questions are posed to engage students’ historical thinking skills: ‘What products might have been transported along the Royal Road from Mesopotamia to Anatolia and from Anatolia to Mesopotamia? How do you think the security of the Royal Road was maintained? Besides trade, how might the Royal Road have influenced civilizations? Just like the example of the Royal Road, there are other trade routes in history and today, and the paths of these trade routes have changed over time. How do you think these changes occurred?’ These questions aim to develop students’ critical thinking, historical context creation, and historical reasoning skills. Additionally, at the end of all activities, a song was played for the students to enjoy, as well as to reinforce the topic and familiarize themselves with the concepts audibly. They were asked to memorize the song and to sing it together.

#### 2.4.6 Activities conducted during the 12th, 13th, and 14th sessions at the Anatolian Civilizations Museum

**Figure 2:**
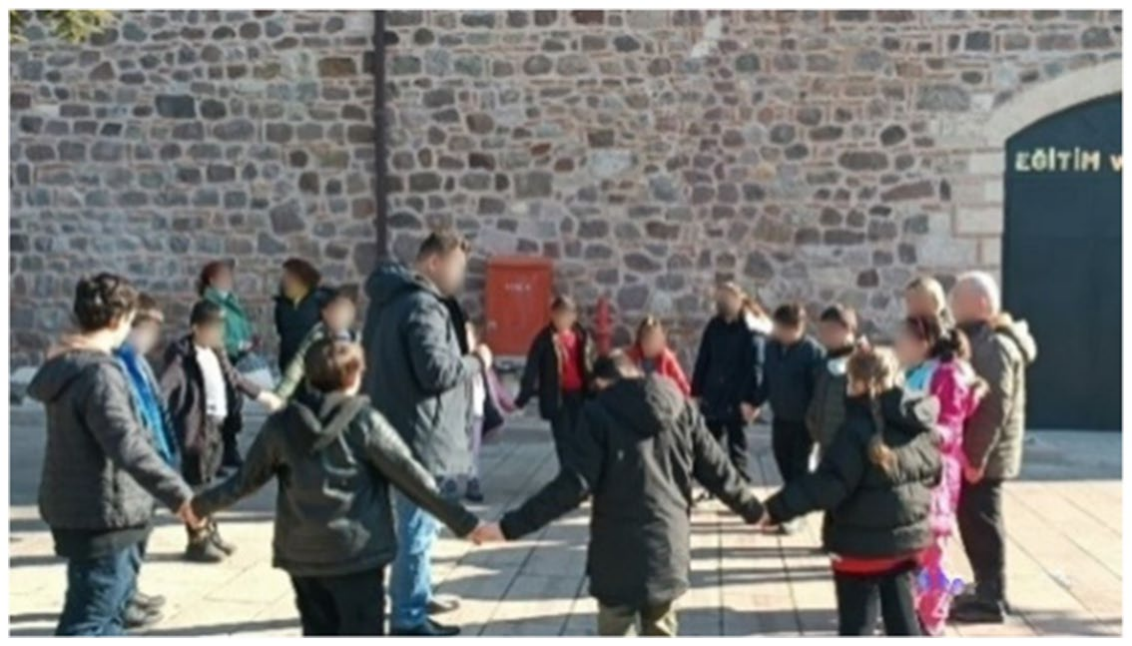
Example of activity in the museum garden.

At the Anatolian Civilizations Museum, students explore prehistoric artifacts related to Anatolian civilizations through the Bongo Art Project^1^^*^ by examining three-dimensional replicas. They try to understand the characteristics of these artifacts by touching, feeling, reading, and listening to descriptions. Students are asked to create a relief work of the artifact that interests them the most using clay dough. In the coin minting workshop, students learn how coins are minted by creating symbolic money. Following activities designed to enhance their historical thinking skills at school, students have the opportunity to apply what they have learned at the Anatolian Civilizations Museum, reinforcing their historical thinking and creativity skills (Figure 2).

## 3 Findings

### 3.1 Findings related to the preliminary interview

#### 3.1.1 Students’ historical era identifications

**Figure 3:**
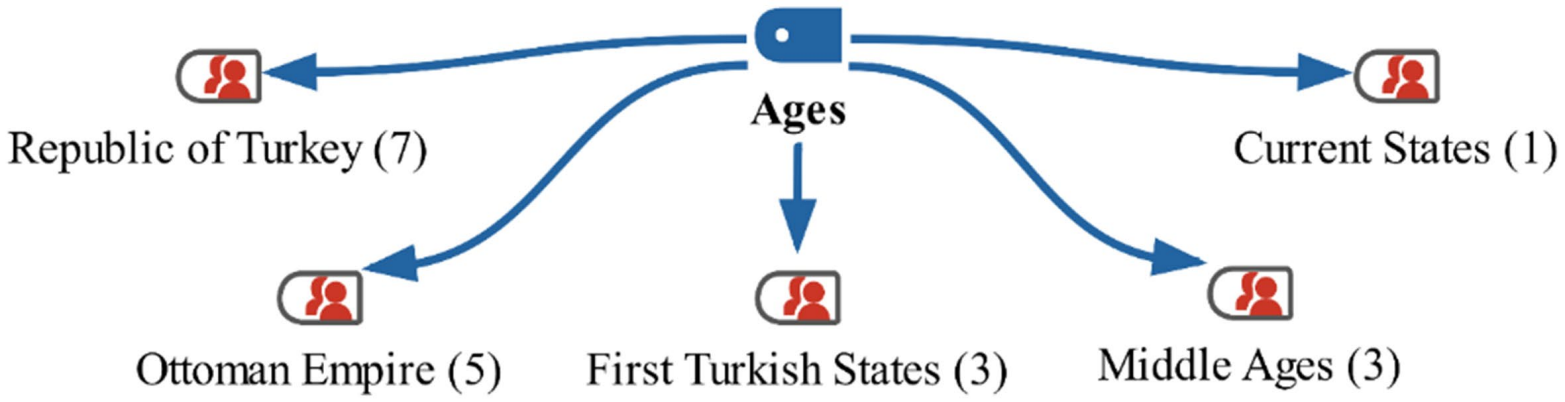
Model for the "ages" category with MaxMaps.

In the interview conducted with the students in the study group before the activities, they were first asked to list the eras starting from the earliest times in history. The students’ responses are shown on the maxmap 1 (Figure 3).

Looking at the students’ responses, it can be said that none of them were able to list the historical eras in a specific order. When examining the maxmap, it is noted that names of states were mentioned more frequently than eras, with only 3 students using the term ‘Middle Ages.’ Some of the students’ responses are as follows:

They can be listed as: First Turkish States, Middle Ages, and Republic of Turkey (*3FS*).

The Middle Ages, Ottoman Empire, and current states come to my mind (*2MS*).

#### 3.1.2 Students’ preferences for historical periods

**Figure 4:**
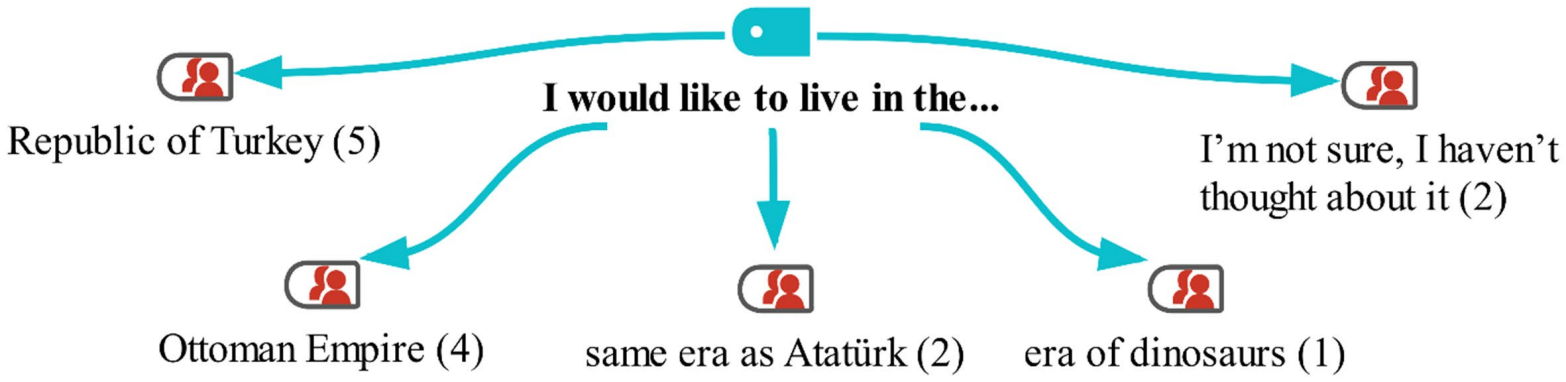
Model for the "I would like to live in the…" category with MaxMaps.

In the interview conducted with the students in the study group before the activities, they were also asked, ‘In which historical period would you like to live? The students’ responses are shown on the maxmap 2 (Figure 4).

Looking at the students’ responses, it can be said that they were unable to articulate historical periods. Five of them expressed a desire to live in the state they currently inhabit. The students mentioned that they feel safe and happy due to the state’s republican governance. Four of the students expressed a desire to live in the Ottoman Empire due to their admiration for the Ottoman sultans. Additionally, some students indicated that they would like to live in the era when Atatürk lived. Finally, one student mentioned wanting to live in the time of dinosaurs due to her interest in them. Two students did not answer this question. Some of the students’ responses are as follows:

I would really like to live in the time of dinosaurs and be able to see them. I've heard many things about them. They are a very different type from the animals we have today. I'm very curious about that period (*4FS*).

Honestly, I would like to live in Turkey, where I am currently. I wouldn’t want to be in a different time or state. I prefer to be here because I feel safer (*7MS*).

#### 3.1.3 Students’ understanding of chronological concepts

In the interview conducted with the students in the study group before the activities, they were also asked to draw a timeline starting from the earliest times in history on Braille embossed paper, illustrating concepts such as ‘era, century, AD, BC, historical period,’ and chronology. Only 2 of the students represented the concepts of BC and AD on the timeline, while one student was able to show modern times. The other students either did not place the mentioned concepts on the timeline or represented them incorrectly. In this context, it can be said that the students lack mastery of chronological thinking skills.

#### 3.1.4 Students’ responses on life in ancient Anatolian civilizations

**Figure 5:**
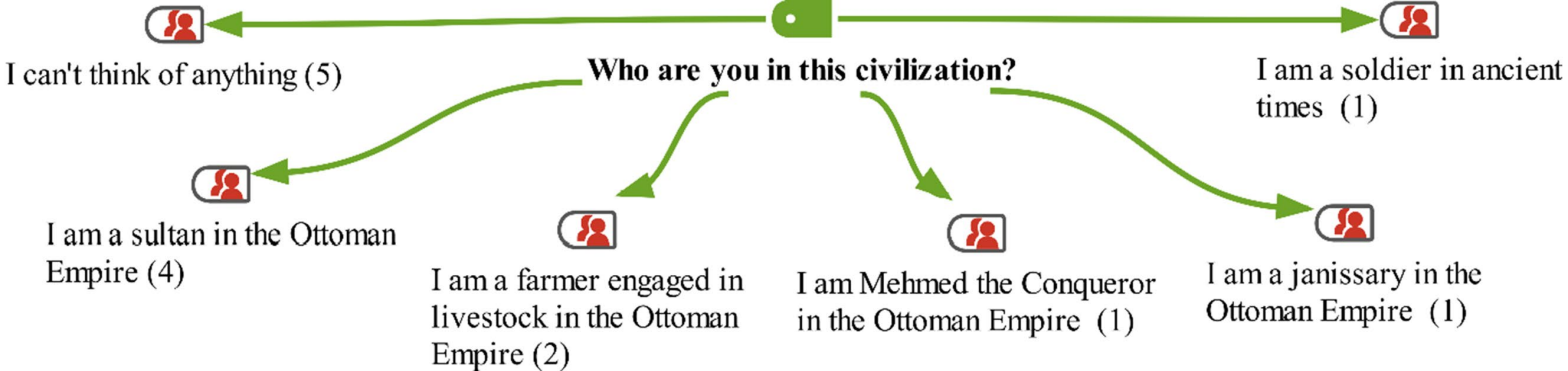
Model for the "Who are you in this civilization?" category with MaxMaps.

In the interview conducted with the students in the study group before the activities, the following instruction was given: ‘Think of an ancient civilization that was established in Anatolia. Imagine that you live in this civilization. Who are you, and what is your occupation? Talk about the name of this civilization, the time period you are in, and its geographical location in Anatolia.’ The students’ responses are shown on the maxmap 3 (Figure 5).

Looking at the students’ responses, it can be seen that 5 students expressed that they could not think of anything for this question. On the other hand, 8 of the other students narrated a scenario related to the Ottoman Empire. In these scenarios, the students imagined themselves as a sultan, a janissary soldier, and a farmer. One student mentioned being a soldier in ancient times but did not specify a particular period; she only described their narrative based on the soldier role they created. As shown on the map, no student mentioned ancient Anatolian civilizations. Some of the students’ responses are as follows:

I am a sultan living in the Ottoman Empire that ruled over Anatolia. My lands extend throughout Anatolia. I live in my grand palace located in Istanbul. I have many servants at my command. I also have palaces in different cities of the country. Sometimes I travel to other cities to listen to the people's problems and learn their wishes. During those trips, I go to my other palaces (*1FS*).

I am a soldier who lived in ancient times. Since I was a child, I always wanted to be a soldier. It is my duty to defend my country and protect it against enemies. Our country is often attacked by enemies. For this reason, being a soldier is a very important duty. Sometimes we go to other countries to fight. During times of peace, we train new soldiers (*4FS*).

### 3.2 Findings related to the final interview

#### 3.2.1 Students’ opinions on educational applications

**Figure 6:**
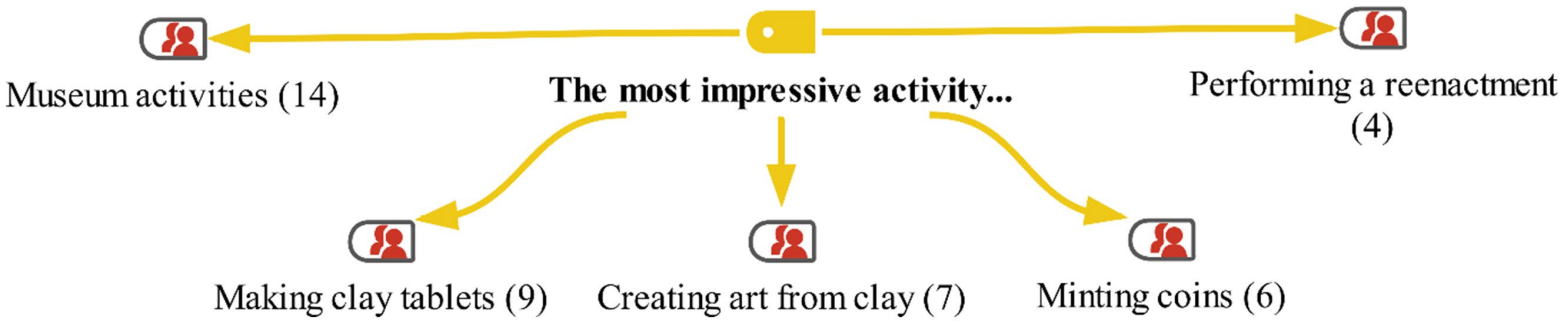
Model for the "The most impressive activity" category with MaxMaps.

**Figure 7:**
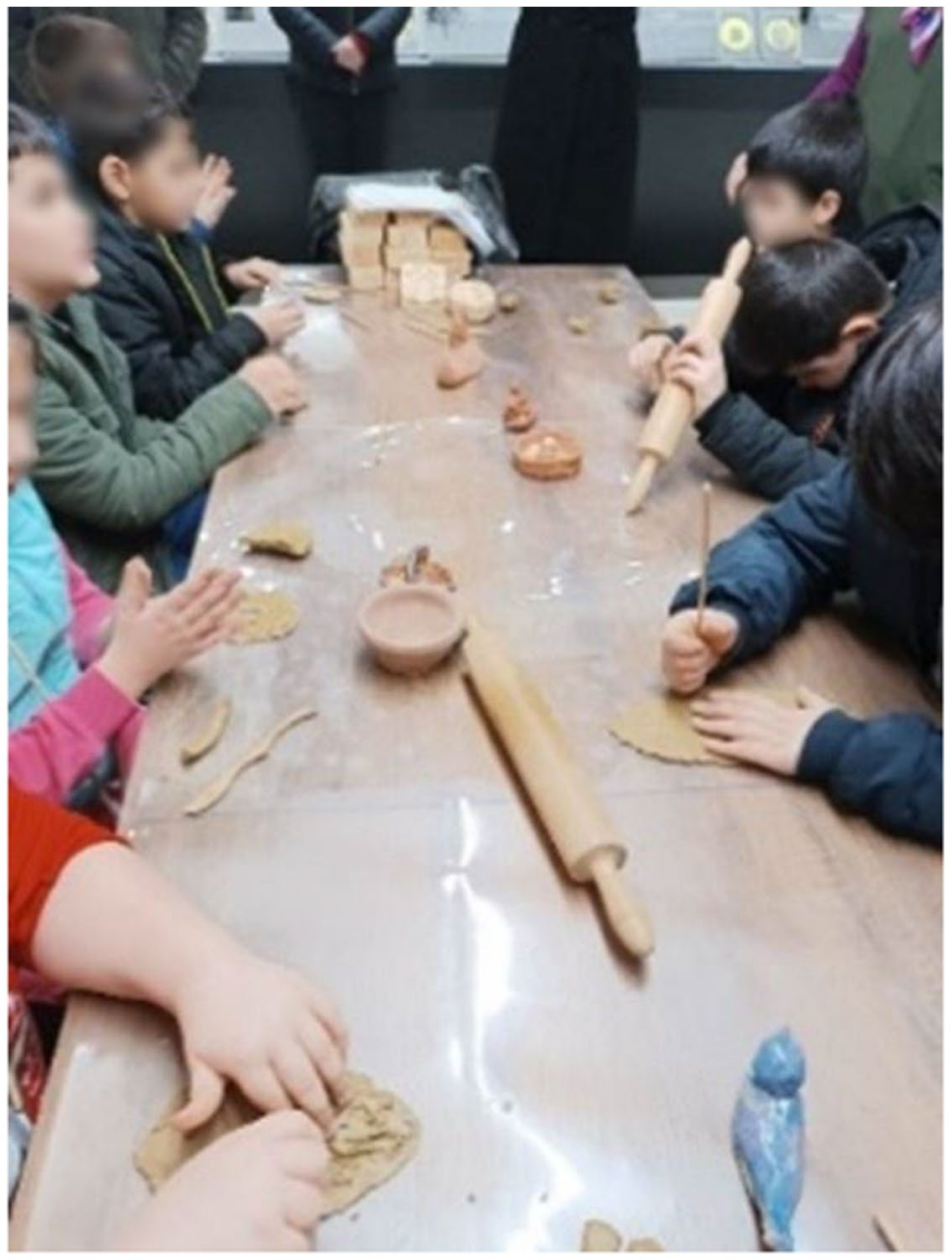
Example of relief making activity in the museum.

**Figure 8:**
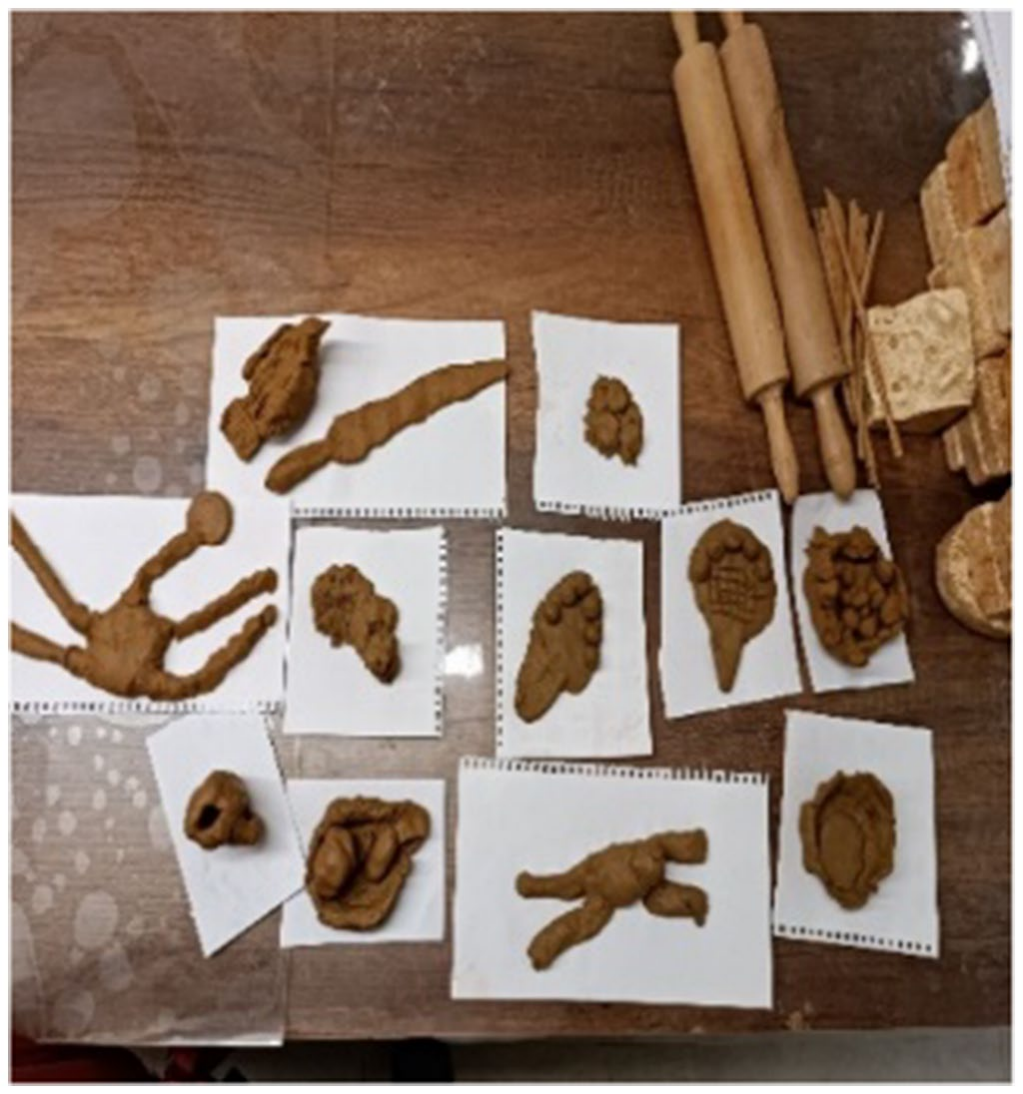
Example of relief making activity in the museum.

**Figure 9:**
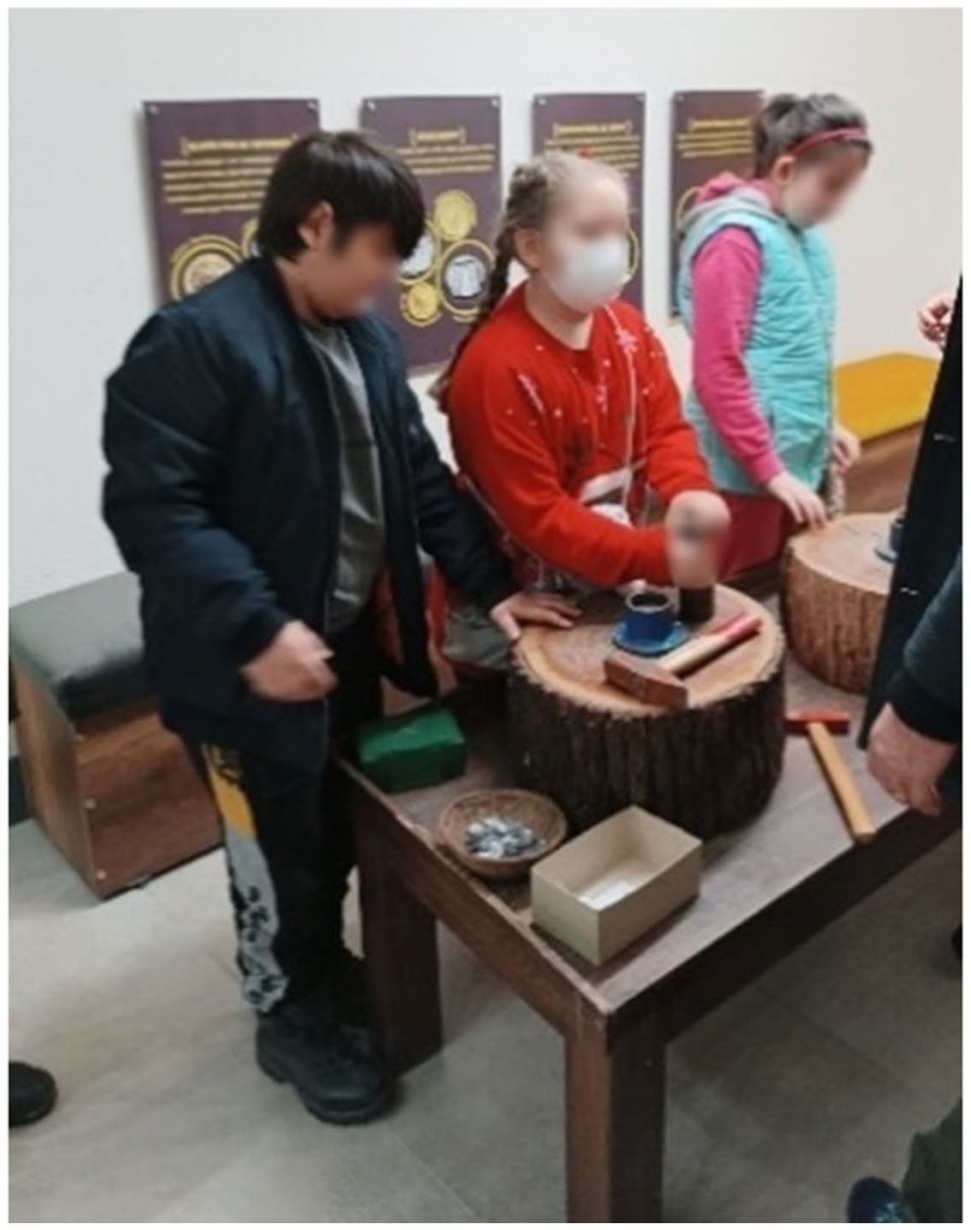
Example of coin minting activity in the museum.

In the interview conducted with the students in the study group at the end of the activities, their thoughts about the applications were first asked. In this context, the students were asked about their opinions on the activities and which activity had the greatest impact on them. The students’ responses are shown on the maxmap 4 (Figures 6–9).

It can be said that all the students in the study group expressed positive opinions about the activities. The students’ responses have been categorized under headings such as ‘it was enjoyable, it was exciting, it was understandable, I wish it were always like this, … ‘Additionally, all the students expressed that they particularly loved the activities they did at the museum. Some of the students’ responses are as follows:

Our teacher had explained these topics, but it was very boring, and I didn't understand anything. I had a lot of fun during the activities you organized, especially at the museum. I learned the topics discussed in class much better at the museum (*1MS*).

The money printing activity excited me a lot. I wondered, ‘Could I get rich by printing money this way? (*5FS*).

It was so much fun trying to make the sitting lion out of clay that I touched in the museum. I wish our classes were always like this… We can’t really see the historical artifacts behind the glass in the museum, so being able to touch the replicas is wonderful (*9MS*).

#### 3.2.2 Highlights of museum activities: student reflection

**Figure 10:**
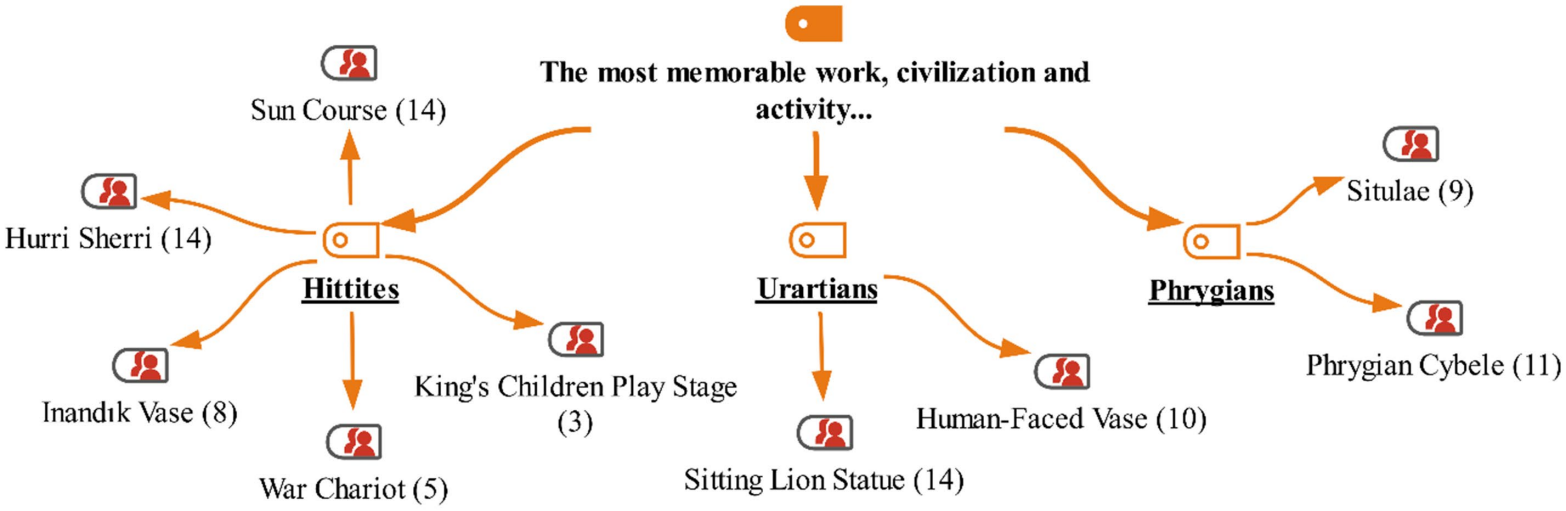
Model for the “The most memorable work, civilization and activity” category with MaxMaps.

**Figure 11:**
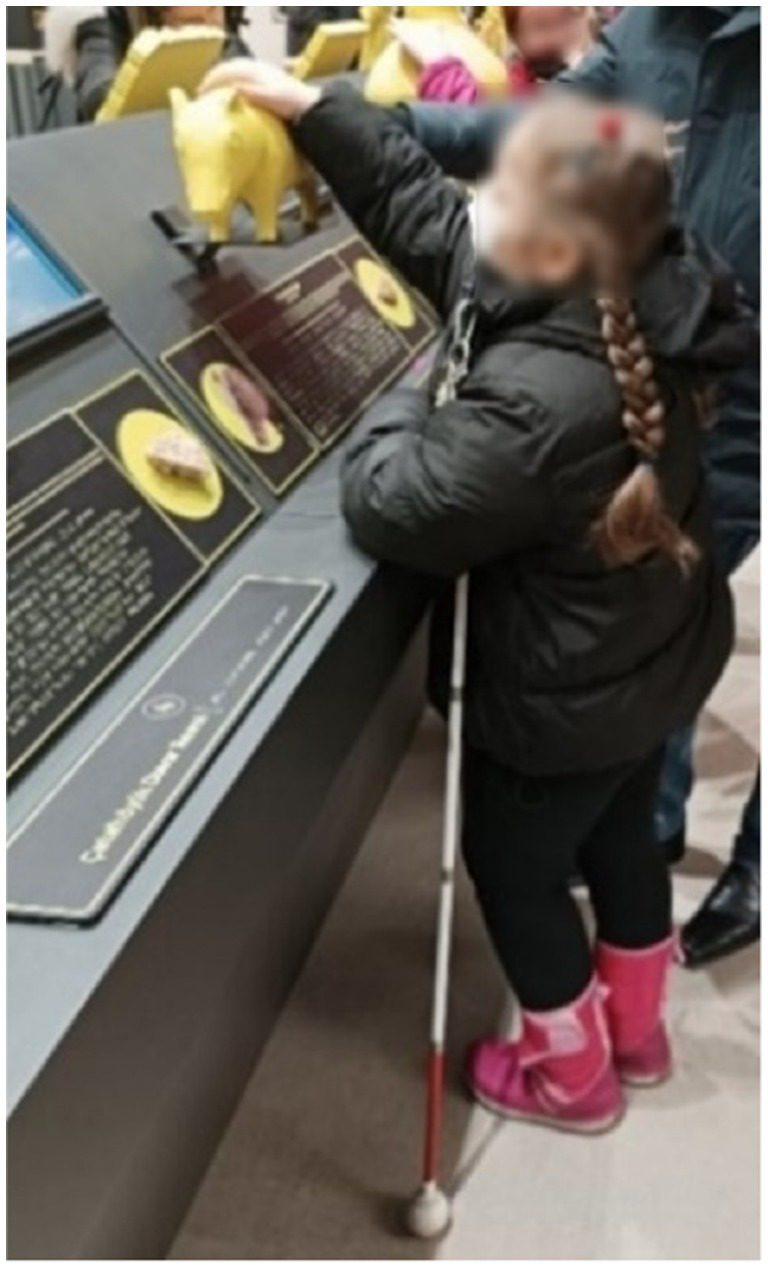
Student examining three-dimensional replica.

**Figure 12:**
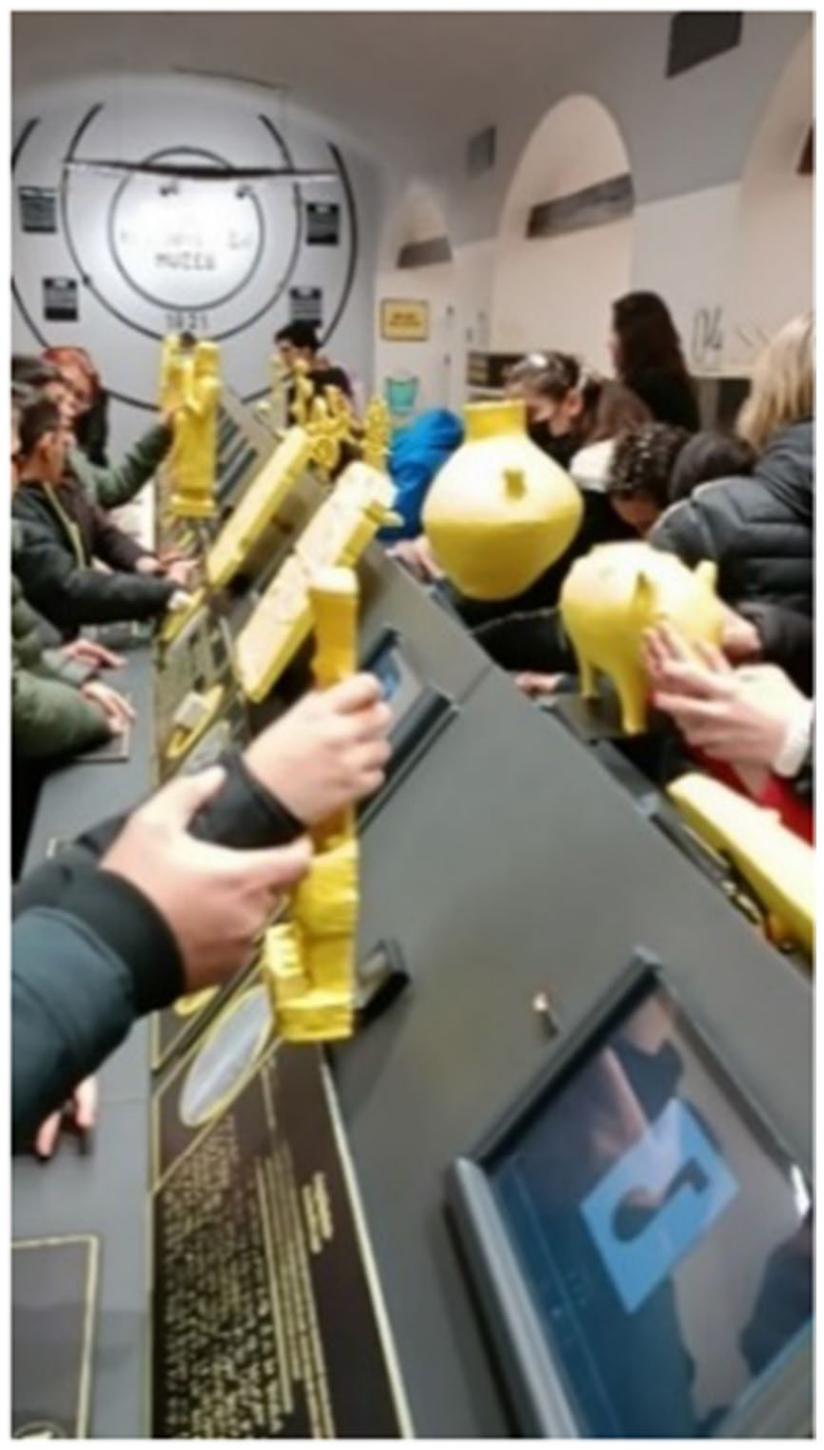
Students examining three-dimensional replica.

At the end of the activities with the students in the study group, they were also asked during the interview which artifacts, civilizations, and activities they remembered the most from the museum events. The students’ responses are shown on the maxmap 5 (Figures 10–12).

Students in the study group (9 students) correctly matched the artifacts with their civilizations. The other 5 students mixed up some artifacts, but when given a hint, they were able to identify the correct civilization. Although the students could not name all the artifacts, they accurately named the civilizations. The activities that the students liked the most were the money printing activity, making clay artifacts, and the activity of reenacting Midas with donkey ears. Some of the students’ responses are as follows:

In the artifacts I touched, there was a human-faced vase. As I touched the vase, I could feel the eyes, nose, and mouth. I realized that the vase has three human faces (*3MS*).

First, I tried to recognize the artifact by touching it with your help. When I first touched the sitting lion statue, I was scared and wondered what it could be. There were two legs in front, and with its mouth open, it had teeth. I touched its face, and the first thing that came to my mind was a wolf, but it wasn’t. When I read the text about the statue, I realized it was a lion (*2FS*).

There were two bulls there. As I touched them, they felt identical with no differences. I could not understand why these two bulls were like that. When I listened to the audio guide describing the statues, I learned that the only difference between the bull statues was the direction of their tails. When I touched them again, I realized that their tails were facing the other way (*5MS*).

When reenacting Midas with donkey ears, the pointed leaves that were attached to my ears were very beautiful, and I really enjoyed portraying Midas (*4FS*).

#### 3.2.3 Students’ Imaginations on Life in ancient Anatolia

**Figure 13:**
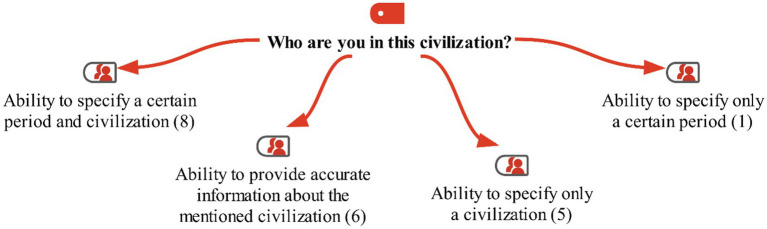
Model for the “Who are you in this civilization?” category with MaxMaps.

At the end of the activities with the students in the study group, the instructions given in the preliminary interview were repeated: ‘Think of an ancient civilization that was established in Anatolia. Imagine that you live in this civilization. Who are you in this civilization, and what is your occupation? Talk about the name of this civilization, the time period it belongs to, and its geographical location in Anatolia. The students’ responses are shown on the maxmap 6 (Figure 13).

When we look at the students’ responses, we can say that their narratives have developed a historical context compared to the preliminary interview. All students, except for one, mentioned the name of a civilization. That one student did not specify a civilization name but expressed that they lived in a civilization in Anatolia during ancient times. The civilizations most frequently mentioned were the Hittites and the Lydians. Some of the students’ responses are as follows:

Hello, I’m Tamuri. I live in the Hittite civilization, which was established in the middle of Anatolia before Christ, with its capital in Hattusa. My father is a farmer, and we produce wheat. We have animals, and my siblings and I help him. We do not even realize how the days pass. While we live in peace, 1 day our relationship with the Egyptians deteriorated, and we went to war. As a result of the war, the Treaty of Kadesh was made, and from that day on, children born were named Peace (*6MS*).

I am a merchant living in the fertile plains of western Anatolia during the Lydian period. I sell rugs, carpets, decorative items, pitchers, and vases. Thanks to the Royal Road, I can sell my goods to other countries. We invented money; before that, there was bartering. They wanted to bring 10 eggs to get a rug, but I would not accept that. They said we could get a rug for whatever we brought, and I would say 10 chickens for a rug. Others could give three chickens for one rug. Trade was very complicated. We solved this problem with money (*2FS*).

#### 3.2.4 Students’ responses to the role of Hittite king

**Figure 14:**
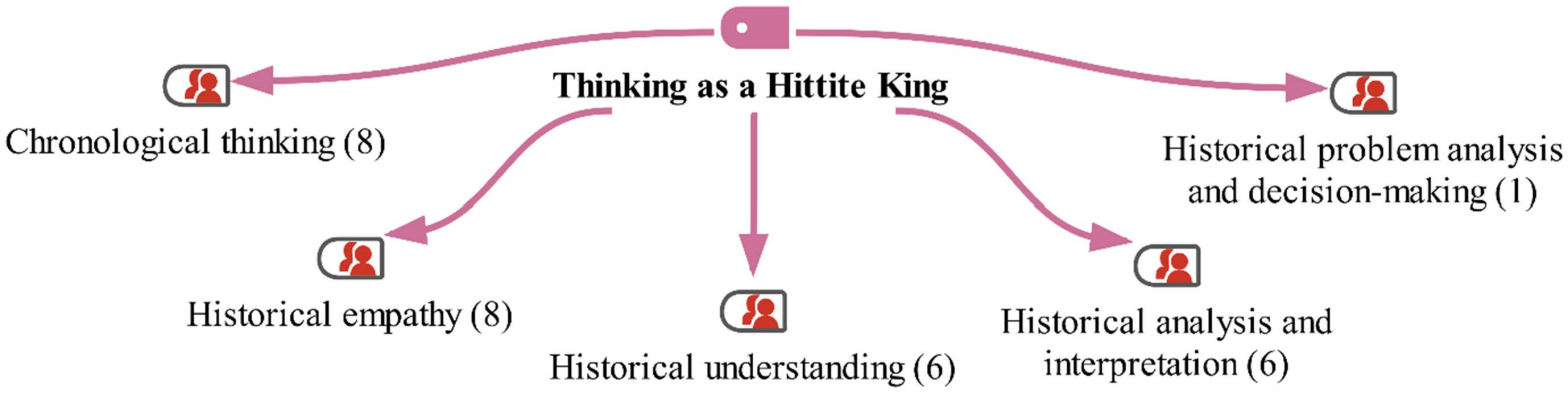
Model for the “Thinking as a Hittite King” category with MaxMaps.

We asked the students to imagine that they were someone living in the civilizations of Anatolia. As a continuation of their previous narratives, they were also asked to think of themselves as the Hittite King and create an example of an annal. The aim of this exercise was to encourage the children to use their historical thinking skills to establish a historical context. The students’ responses are shown on the maxmap 7 (Figure 14).

In the examples of annals created by the students, the texts generally demonstrate that the children were able to achieve historical perspectives and contexts. However, in some texts, although the development of the students’ historical thinking skills is evident, some incorrect historical contexts were also observed. Nevertheless, it can be said that, overall, the students have acquired historical thinking skills, particularly in chronological thinking and historical empathy. Some of the students’ responses are as follows:

I am Hutti^1^, the Hittite king. I ascended to the throne last year^.2^ To ensure my people live comfortably, I provided them with food and drink^3^. To establish justice, I made additions to the existing laws^4^. I defeated the Hurrian state, which acted against me and invaded our lands without permission, in battle. I took many captives. The sun god protected me. I held festivals for the sun god. I will fulfill any tasks assigned to me by the sun god^5^… (*5FS*).

*1. Historical empathy, 2. Chronological thinking, 3, 4. Historical understanding 5. Historical analysis and interpretation.*

In the fifth year^1^ of my reign, I worked with Queen Hudepapa to ensure the peace of our state. I decided to stop bartering and to start using money^2^. Thanks to money, our country became wealthy^3^. To maintain the peace of our country, I made peace treaties with neighboring states^4^. However, other envious and hostile nations declared war on me. Many of my soldiers died in the war^5^. I pleaded with the gods to save my country and me from this dire situation, but they did not help me. The gods must be angry with me for introducing Money^6^ … (*8MS*).

1. Chronological thinking, 2. Historical problem analysis and decision-making, 3, 4. Historical understanding, 5, 6. Historical analysis and interpretation.

#### 3.2.5 Students’ analysis of the Kadesh war

**Figure 15:**
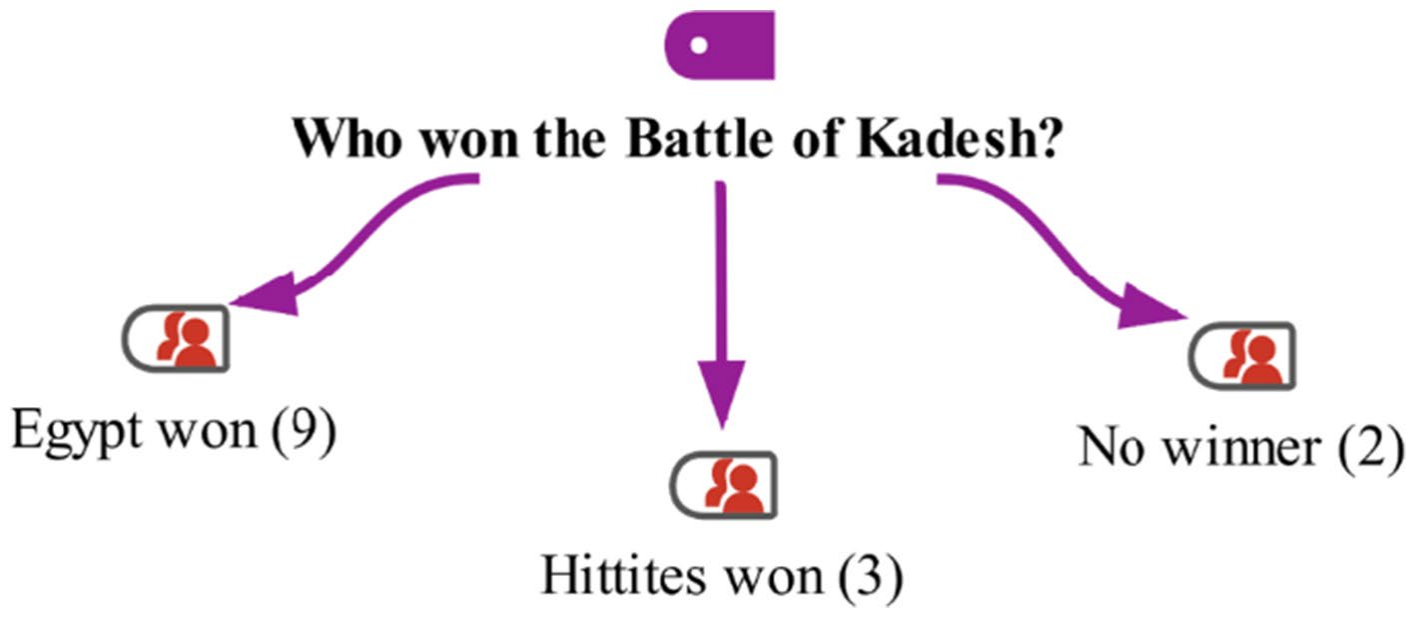
Model for the “Who won the Battle of Kadesh?” category with MaxMaps.

At the end of the activities with the students in the study group, the Kadesh War discussed during the events is brought up. The students read primary sources related to the Kadesh War, including Egyptian and Hittite texts as well as the articles of the Treaty of Kadesh. Then, the question ‘Who won the Kadesh War?’ is posed to them. The students’ responses are shown on the maxmap 8 (Figure 15).

The students developed their ‘historical analysis and interpretation skills’ by establishing cause-and-effect relationships between historical events and comparing the similarities and differences of historical texts containing various opposing views, using critical thinking skills to reach a judgment about who won the war. Some of the students’ responses are as follows:

Because the war is described in more detail and dramatically in the Egyptian texts, I believe the Egyptians won the war (*6MS*).

I believe the Hittites won. Because they had war chariots, which are very important in battle (*3FS*).

There is no winner of the war. Because despite both states claiming that they won, the terms in the Treaty of Kadesh appear to be equal. The leaders of both states referred to each other as brothers and stated that they would help each other in case of an attack from other states (*3MS*).

#### 3.2.6 Students’ timeline drawings

At the end of the activities with the students in the study group, they were asked to draw a timeline starting from the earliest times in history on Braille embossed paper, illustrating the concepts of ‘era, century, BC, AD, historical period’ and chronology’. The students generally placed the concepts and chronology correctly. Only 2 students needed help while placing them. In the preliminary interview, the students were also asked to place the concepts and chronology on the timeline. Compared to the preliminary interview, the students were successful in placing the required concepts and chronology. In this regard, it can be said that the students have mastered chronological thinking skills.

To evaluate the findings generally, it can be said that the visually impaired students in the study group began to learn historical concepts, events, and civilizations more effectively through hands-on activities and interactive museum visits. A significant development was observed, especially in skills such as contextualizing historical concepts, thinking in a historical context, evaluating past events with empathy, and analytical thinking. By the end of the educational process, the students had gained a stronger historical perspective, developed the ability to make comparisons between different historical periods, and acquired the skill to create historical context. As a result, it can be said that visually impaired students expanded their historical perspective and developed a more conscious and critical point of view.

## 4 Conclusion

When we evaluate the results of this study, which aims to teach history to a group of visually impaired middle school students to develop their historical thinking skills and creativity, it can be said that the students in the study group had the opportunity to enhance their historical thinking skills and creativity within the framework of the 5th Grade Journey to History unit.

Before the research, a preliminary interview was conducted with the students in the study group regarding the unit topics, as the visually impaired students had already covered the Journey to History unit in their Social Studies class. In this interview, it was observed that none of the students could list the historical periods in a specific order, and they did not have a good grasp of the concepts of historical time and chronology. Instead of naming periods, they mentioned the names of states, and only 3 students referred to the term ‘Middle Ages.’ Additionally, when the students were asked which era they would like to live in, they again mentioned a specific state or period without stating the name of the era. It was also observed that, within the framework of the Journey to History unit (Evirgen et al., 2021), the students were unable to place the concepts of ‘era, century, BC, AD, historical period’ on the timeline they studied. Additionally, in the preliminary interview, the students were asked to imagine living in the ancient civilizations of Anatolia BC and to create a narrative. Ten of the students could not create this narrative. The other students, however, provided narratives set in a more recent period by mentioning the names of states instead of referring to the ancient civilizations of Anatolia.

As a result, it can be said that the visually impaired students in the study group did not fully learn the concepts and topics covered in the 5th Grade Journey to History unit during the preliminary interview. Additionally, in the discussion held with the teacher before the application, the teacher’s statement (due to the impossibilities at the school, the unit was taught in a teacher-centered manner, relying on the textbook) supports the conclusion that the students did not fully learn this unit. In the literature, Çelikkaya and Kürümlüoğlu (2019) found in their study with middle school students that only one student was able to answer the question regarding the chronological order of historical eras from the past to the present. In the study, they also noted that a large majority of the students provided incorrect answers or left the question about identifying events that occurred BC and AD blank. Similarly, in the study by Çiviler (2019), it was revealed that 7th-grade students have low levels of historical thinking skills and perception of historical concepts in Social Studies class. In the study conducted by Pala and Şimşek (2016), it was also found that students have a low level of knowledge regarding ancient history (such as the founding and collapse dates of civilizations established in Anatolia). Additionally, the study by Safran and Şimşek (2006) indicated that three-quarters of the students could not demonstrate processing skills related to BC and AD.

Despite the developmental delays experienced by visually impaired individuals, most process cognitive information similarly to sighted individuals (Warren, 1994). The abilities of visually impaired students are not different from those of regular students (Kholoud et al., 2015; Abrahamson et al., 2019). The differences between visually impaired and sighted students emerge in their methods of information gathering; however, these differences diminish in cognitive structure and processing (Hollins, 2000). However, since learning at school is largely dependent on vision, students with this disability often face academic challenges (Kumar et al., 2001). In this study, it can be said that an effective learning atmosphere was provided for visually impaired students to minimize the difficulties they experience in history education. In this learning environment, well-designed questions aimed at developing historical thinking skills, as well as activities such as evidence-based learning, discussion, analogy, role-playing, simulation, time travel, brainstorming, and creative writing, were applied. The literature emphasizes that in the education of visually impaired students, it is essential to select appropriate methods, techniques, or strategies for relevant topics to help them understand abstract information in their cognitive processes. It highlights the need for alternative learning materials and, in summary, that the learning environment should be adapted to their needs (Özyürek, 1998; Koenig and Holbrook, 2000; Lyndon Gillon and Young, 2002; Şahin and Yörek, 2009; Evans and Douglas, 2008; Kapucu and Kızılaslan, 2022; Şahin-Kölemen and Akgün, 2022). Additionally, Akbaba (2020) emphasized the importance of well-organizing learning and teaching processes for the development of historical time and chronology thinking. In this respect, the study also shows similarities with other studies in the literature conducted with visually impaired students in different fields (Simon et al., 2010; Bayram et al., 2015; Okcu et al., 2022).

In the interviews conducted at the end of the application process in the research, it was observed that the students in the study group showed development in their ability to use historical time concepts and chronological knowledge correctly compared to before the application. They were able to place the concepts of ‘era, century, AD, BC, historical period’ on the Brille embossed timeline. It was noted that they had improved in using historical time and chronology concepts accurately, correctly identified historical periods, recognized civilizations that lived in Anatolia during the pre-Christian eras and matched the artifacts of these civilizations with their respective cultures. When looking at the literature, it is similarly noted that Çalışkan and Varlıkgörücüsü (2020) used timelines in their studies and demonstrated that these timelines are an effective tool for helping students develop skills in perceiving time and chronology. Additionally, in Çiviler’s (2019) study, it was found that through various activities in the 7th-grade Social Studies class, students improved their abilities to distinguish between historical facts and interpretations, perceive time and chronology, develop historical empathy, and recognize stereotypes. The action research concluded that students’ historical thinking skills had developed as a result. Many studies on the teaching of historical time regarding classroom practices show that timelines are important for developing students’ understanding of historical time (Alleman and Brophy, 2003; Hodkinson, 2003; Dawson, 2004; Şimşek, 2007; Barton, 2011; Cooper, 2012; Blow et al., 2012; Sağlam et al., 2015; Altun and Kaymakcı, 2016; Groot-Reuvekamp et al., 2018; Çalışkan and Varlıkgörücüsü, 2020; Aktın and Karaçalı Taze, 2023). Additionally, studies conducted in other fields (Neely, 2007; Kızılaslan et al., 2020; Okcu and Sözbilir, 2016; Kadir and Şimşek, 2021; Rule, 2011; Wild et al., 2013; Chinthaka and Abeygunawardhana, 2018; Götzelmann, 2018; Karbowski, 2020) show similarities in that lesson activities designed for the needs of visually impaired students have successfully facilitated concept teaching.

In the study, in addition to recognizing and accurately expressing historical periods, students demonstrated their creativity by imagining narratives in which they lived in Anatolian civilizations during pre-Christian eras, using historical knowledge correctly. In their narratives, students imagined themselves living in one of these civilizations and introduced the civilization and the conditions of that era. They were also asked to continue their narratives by imagining themselves as the Hittite King and to create an example of an annal. It can be said that in these annal examples, the children generally established historical perspectives and contextualization. Additionally, students were asked to analyze the causes and effects of historical events and to comment on who won the Battle of Kadesh. By comparing the similarities and differences of historical texts containing various opposing viewpoints, students were able to develop their ‘historical analysis and interpretation skills’ and, using critical thinking skills, reach a judgment about who won the war. The narratives created by the visually impaired students in the study group indicate that they achieved the learning outcomes of the 5th-grade ‘Journey to History’ unit, particularly in historical empathy, historical thinking skills, and creativity. It can be said that the annal activity and the Battle of Kadesh activity conducted with the students were aimed at evaluating primary evidence. Many sources in the literature point to the importance of teaching history using primary evidence (Scott and Kirk, 2010; Özbaş, 2010; Culminas-Colis et al., 2016; Cox, 2018; Bekret, 2019; Çiftarslan, 2019; Çiviler, 2019; Lee, 2023). Similarly, in their study, De Leur et al. (2017) asked students to write texts by vividly imagining themselves or someone from the past. In this study, which encourages students to imagine the emotions of historical actors, they facilitated students’ historical empathy by reconstructing the feelings of people from the past. In his study, Barton (1997) had students write compositions from the perspective of people who lived in the past and make observations about the changes in daily life between the past and the present. His work revealed that students were able to critically examine sources compared to before.

In the interviews conducted at the end of the application process, the students in the study group were asked for their opinions about the activities carried out. The students expressed that they enjoyed their experiences and found the process enjoyable. All participants shared positive feedback about the process and agreed that their favorite activities were the ones conducted in the museum. Similarly, Marovah and Ncube (2023) emphasized that incorporating museums into middle school history education has the potential to enhance motivation among students. In their study, Demir and Köksal Akyol (2022) found that visually impaired children enjoyed the museum visit, noting that the opportunity for tactile engagement was a significant factor in their enjoyment of the trip. Additionally, Danacı Polat and Buyurgan (2022) stated in their study that visually impaired children particularly loved the tactile activities during the museum visit and felt that the museum experience exceeded their expectations. Kouseri (2019) also found that students perceived the museum as a different, welcoming, and open space, describing their experience of studying history there as inspiring, engaging, and enjoyable, in contrast to the classroom environment. Jo et al. (2016) demonstrated in their study that 3D printing technology, which allows visually impaired students to appropriately feel historical images, maps, or artifacts, helps these students successfully understand the content being taught. Argyropoulos and Kanari (2015) shared in their study the benefits of allowing exploration through touch, citing a participant’s words after a museum activity: ‘Touching the exhibits in the museum is magical, a mystery. I will never forget this experience.’ In their study, Buyurgan and Demirdelen (2009) found that allowing students with visual impairments to touch replicas and copies of certain artifacts in the museum made them eager and excited to learn throughout the process, leading to high learning potential. Hooper-Greenhill (1999) also mentioned that touching and handling objects in the museum can create excitement, increase learning motivation, and even initiate the learning process.

This study highlights the importance of museum activities for students learning about a specific historical period, as it enables them to recognize the civilizations that lived during that time and the traces they left behind. This is crucial because visually impaired students are tactile and kinesthetic learners who need touch to learn the content (Şahin and Yörek, 2009). Aktaş and Durak (2021) concluded in their study that museum visits are important educational settings for revealing and developing students’ time and chronology skills. Similarly, Martinko and Luke (2018) stated that hands-on experiences with objects in museums can provide the necessary structures for children to engage in historical thinking, suggesting that children demonstrate multiple examples of historical thinking in applied history contexts. In a study conducted in a museum with middle school students, Wallace-Casey (2017) noted that participants recognized the problematic nature of historical inquiry and expressed that students could physically engage with the past by moving around in the museum. Artar (2010) explained why museums and education should be combined, emphasizing aspects such as experiential learning, fulfilling creativity and curiosity, ensuring lasting learning, and bringing education from the abstract to the concrete. Additionally, Dyson (2010) suggested in the literature that children can understand history in museums, particularly through playful, imagination-based experiences.

Considering the results of the research, it can be said that there is a need for examples aimed at teaching history to visually impaired students and developing their historical thinking skills. In this regard, it is necessary to create a classroom atmosphere tailored to the needs of visually impaired students in history teaching and to select methods and techniques that will contribute to their learning, as well as to develop appropriate materials. Additionally, more frequent visits should be made to museums where artifacts are suitable for touch, allowing visually impaired students to learn through direct interaction with primary sources.

For future research, more comprehensive studies need to be conducted on how the historical thinking skills of visually impaired students can be developed more effectively. Additionally, exploring innovative tools and technologies to enhance the accessibility of materials used in history education and redesigning curricula to better address the needs of this student group is crucial. In this process, equipping teachers with special education and pedagogical skills to work with visually impaired students will be an important step towards promoting equitable approaches in education. Developing curriculum designs that create experience-based learning opportunities and make history teaching more efficient for visually impaired students will also contribute to educational policies in this field.

## 5 Limitations

In this study conducted with visually impaired students within the framework of the 5th-grade ‘Journey to History’ unit, the inability to apply all stages of the indicators for historical thinking skills is the most notable limitation of the study. This is because students need to access primary sources themselves to develop historical thinking skills. They are expected to find, read, and write about the sources they understand. In this study, these indicators could only be applied by the researchers reading to the students. Additionally, the inability of the students in the study group to directly access visual elements caused the process to rely on the researchers’ descriptions, leading to difficulties for the students in concretizing the material during the application process. In addition, it is still very difficult for visually impaired students to access touch-feel-audio materials on many history topics. This difficulty is perhaps the most important limitation for teaching history to the visually impaired.

## Data Availability

The original contributions presented in the study are included in the article/supplementary material, further inquiries can be directed to the corresponding author.

## Footnotes

^1^ https://www.youtube.com/watch?v=ul8K5l4wxbk

^*^ https://gorenkalpler.org/bongo-art-project-ile-muzeler-engelliler-icin-eriselibilir-hale-geliyor
